# Levantilides A and B, 20-Membered Macrolides from a *Micromonospora* Strain Isolated from the Mediterranean Deep Sea Sediment

**DOI:** 10.3390/md9010098

**Published:** 2011-01-14

**Authors:** Andrea Gärtner, Birgit Ohlendorf, Dirk Schulz, Heidi Zinecker, Jutta Wiese, Johannes F. Imhoff

**Affiliations:** Kieler Wirkstoff-Zentrum (KiWiZ) at the IFM-GEOMAR (Leibniz Institute of Marine Sciences), AmKiel-Kanal 44, 24106 Kiel, Germany; E-Mails: agaertner@ifm-geomar.de (A.G.); bohlendorf@ifm-geomar.de (B.O.); dschulz@ifm-geomar.de (D.S.); heidi.zinecker@uni-bonn.de (H.Z.); jwiese@ifm-geomar.de (J.W.)

**Keywords:** macrolide, *Micromonospora*, deep sea, natural product, marine

## Abstract

Two new 20-membered macrolides, levantilide A and B, were isolated from the *Micromonospora* strain M71-A77. Strain M71-A77 was recovered from an Eastern Mediterranean deep-sea sediment sample and revealed to produce the levantilides under *in situ* salinity of 38.6‰. The chemical structures of the levantilides were elucidated on the basis of different one- and two- dimensional NMR experiments. Levantilide A exhibits a moderate antiproliferative activity against several tumor cell lines.

## 1. Introduction

The deep sea is an extreme environment which is still marginally investigated and harbors a great variety of bacteria that have, so far, not been cultivated. Bacteria which live in the deep sea need to adapt to the specific environmental characteristics such as high hydrostatic pressure, low temperature and only occasional nutrient supply. These constraints quite likely determine the phylogenetic diversity of the deep-sea bacterial communities and also affect the secondary metabolite production of these bacteria. Therefore, deep-sea bacteria are considered as a promising source for the discovery of new natural products. Marine members of *Actinobacteria* are highly potent producers of interesting compounds [1–5] as was already shown for their terrestrial counterparts [6]. Only recently, two strains of *Streptomyces* sp. from the Atlantic ocean deep-sea sediment were shown to produce the two new natural products caboxamycin and albidopyrone [7,8].

With special focus on the discovery of new natural products, we selectively isolated *Actinobacteria* from the deep-sea sediment of the Eastern Mediterranean Sea (the so-called Levantine Sea). This environment is characterized by a relatively high bottom temperature of 13–14 °C, salinity values of approximately 38–39‰, high hydrostatic pressure (440 bar at the sampling site) and an extreme depletion of nutrients [9]. Among the isolated bacteria, strain M71-A77 produced two new macrolides named levantilides A (**1**) and B (**2**), which will be described in this paper.

## 2. Results and Discussion

Strain M71-A77 was isolated from the Eastern Mediterranean deep-sea sediment (4400 m) and revealed 99.3% 16S rRNA gene sequence similarity to *Micromonospora auratinigra* DSM 44815^T^ (AB159779). Analyses of the culture extract of this strain (incubated in liquid soja-peptone medium) by HPLC-DAD-MS led to the detection of two unknown 20-membered macrolides, levantilide A (**1**) and B (**2**), with detected masses of *m/z* 508 and *m/z* 506, respectively. Subsequent cultivation of the strain in larger scale (10 L) led to the isolation of the levantilides as colorless solids.

**Figure:**
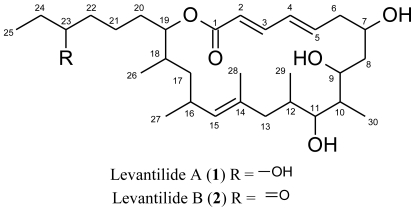


**Figure 1:**
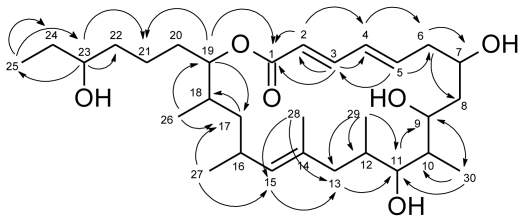
Selected HMBC correlations relevant for the structure elucidation of **1**.

Levantilide A (**1**) had a measured molecular mass of *m/z* 531.3676 [M + Na]^+^ (calculated for C_30_H_52_NaO_6_ 531.3656), which yielded the molecular formula C_30_H_52_O_6_ and implied five degrees of unsaturation. The structure elucidation of the compound was mainly based on one- and twodimensional NMR spectra of **1**. The respective ^13^C NMR spectrum displayed 30 distinct signals (Table 1) which were consistent with the deduced molecular formula. The carbon resonances gave evidence of one carbonyl carbon (δ_C_ 166.1 ppm), one olefinic quaternary carbon (δ_C_ 132.8 ppm), five olefinic methine carbons (δ_C_ 120.4 ppm to 143.8 ppm), five methine carbons adjacent to oxygen atoms (δ_C_ 67.2 ppm to 77.4 ppm), four further methine carbons (δ_C_ 29.4 ppm to 41.0 ppm), eight methylene groups (δ_C_ 22.0 ppm to 40.9 ppm) and six methyl groups (δ_C_ 10.1 ppm to 21.5 ppm). The HSQC spectrum allowed all carbon resonances to be unambiguously assigned to the resonances of their directly attached protons. The final planar structure of the molecule was deduced from COSY and HMBC spectra (Figure 1). With a shift of 166.1 ppm, the carbonyl carbon C-1 was very likely to belong to a conjugated ester function. HMBC correlations from H-3 (δ_H_ 7.06) and H-2 (δ_H_ 5.78) identified the adjacent methine groups. H-2 to H-5 (δ_H_ 6.08) were all olefinic protons which coupled to each other. H-5 showed HMBC correlations to C-3 (δ_C_ 143.8) as well as to C-6 (δ_C_ 39.6), a methylene carbon, and C-7 (δ_C_ 67.2), a hydroxylated methine. H-7 (δ_H_ 3.98) showed COSY correlations to H-6 as well as to the methylene group CH_2_-8 (δ_C_ 33.4, δ_H_ 1.51; 1.16). Next to CH_2_-8, there followed four methine groups, CH-9 to CH-12, each of them substituted with either a hydroxy group, CH-9 (δ_C_ 67.4, δ_H_ 3.92) and CH-11 (δ_C_ 75.3; δ_H_ 2.98), or a methyl group, CH-10 (δ_C_ 41.0; δ_H_ 1.68) and CH-12 (δ_C_ 32.1 and δ_H_ 1.24), which could be unequivocally proven by their shifts, COSY and HMBC correlations. Not only did the COSY correlations of the methyl groups CH_3_-29 (δ_C_ 17.7 and δ_H_ 0.58) and CH_3_-30 (δ_C_ 11.1 and δ_H_ 0.86) connect them to the methine groups CH-12 and CH-10, but their HMBC correlations also gave further evidence of the positions of the neighboring carbons and secured the sequence from CH-9 to CH-12. Furthermore, a methylene group CH_2_-13 (δ_C_ 40.1, δ_H_ 1.87 and 1.51) was shown to connect the methine groups CH-9 to CH-12 to the quaternary olefinic carbon C-14 (δ_C_ 132.8). Therefore, the last double bond evidently was located between C-14 and C-15 (δ_C_ 132.7). The corresponding proton H-15 (δ_H_ 4.76) showed long range couplings to C-17 (δ_C_ 40.9), C-13, C-16 (δ_C_ 29.4), C-27 (δ_C_ 21.5) and C-28 (δ_C_ 17.0) and coupled to H-16 and H_2_-13. CH_2_-17 (δ_H_ 1.34 and 1.05) formed the junction between the methyl-bearing methines CH-16 and CH-18 as indicated by HMBC correlations from H-17 to C-18 (δ_C_ 33.4), C-26 (δ_C_ 17.5) and C-27. The substructure was further supported by COSY correlations of the same proton signal (H-17) to the resonances of H-16 (δ_H_ 2.59) and H-18 (δ_H_ 1.75). CH-19 (δ_C_ 77.4; δ_H_ 4.71), the methine adjacent to CH-18, closed the macrolide ring as proven by its HMBC correlation to C-1 and connected it to the side chain of the molecule by correlations to the methylene groups CH_2_-20 (δ_C_ 27.9; δ_H_ 1.52 and 1.47) and CH_2_-21 (δ_C_ 22.0; δ_H_ 1.30). After the carbonyl group and the three double bonds, one degree of unsaturation still had to be accounted for, which was accomplished by the closure of the ring. Analysis of the NMR spectra gave evidence of a 3-hydroxy-hexyl-side chain. All double bonds, Δ^2,3^, Δ^4,5^ and Δ^14,15^, were determined to be *E*-configured. For the double bonds Δ^2,3^ and Δ^4,5^, the configuration was deduced from the ^3^*J* coupling constants of approximately 15 Hz. Δ^14,15^ is a trisubstituted double bond, therefore the NOESY spectrum had to be consulted. As H-15 showed NOESY-correlations to H_2_-13, but not to H_3_-28, this double bond, too, had to be *E*-configured. Thus, the planar structure of levantilide A could be unambiguously delineated from the spectroscopic data.

The derivative, levantilide B (**2**), showed a mass difference of 2 amu in the HPLC-DAD-MS measurement, which indicated one additional double bond, which for example can be observed, when a hydroxy-group is replaced by a carbonyl function, as it was the case here. Already in the ^1^H NMR spectrum it was obvious that all signals belonging to protons of the macrolide ring were identical in both molecules (see Table 2). However, significant differences could be observed for the signals of the side chain. Analysis of the data showed that the methine group CH-23 was no longer present in levantilide B. Instead of it, an additional signal of a carbonyl carbon appeared, its shift of 210.5 ppm proving it to be a ketone. As a consequence of the presence of a carbonyl group instead of a methine in position 23, the signal of H_2_-24 was no longer a multiplet, but appeared as a quartet as it only coupled with the methyl group CH_3_-25. Thus, the planar structure of **2** was established as depicted.

The levantilides are macrolides with a 20-membered lactone ring. From a biosynthetic point of view macrolides are typical type I PKS products with very well studied biosynthetic pathways. From the structures of the levantilides, a very simple assembly of a propionate starter unit, five further propionate building blocks and altogether six acetate building blocks can be deduced.

Cytotoxicity tests of **1** revealed antiproliferative activities against gastric tumor cells GXF 251L (IC_50_ = 40.9 μM), lung tumor cells LXFL 529L (IC_50_ = 39.4 μM), mammary tumor cells MAXF 401NL (IC_50_ = 28.3 μM), melanoma tumor cells MEXF 462NL (IC_50_ = 48.6 μM), pancreas tumor cells PAXF 1657L (IC_50_ = 20.7 μM) and renal tumor cells RXF 486L (IC_50_ = 52.4 μM). Antimicrobial activity against the bacteria and fungi in the test panel were not observed for compounds **1** and **2**.

Members of the *Actinomycetes* are well known to produce macrolide antibiotics [10]. Micromonospolides, mycinamicins, megalomicin, rosamicin and juvenimicins are e.g., macrolide antibiotics produced by members of the genus *Micromonospora*, but they all differ in the size of the macrolide ring from the levantilides [11–15]. The levantilides are 20-membered macrolides without an attached sugar and are, for example, related to the cytotoxic macrolides amphidinolide A and U [16,17] as well as to iriomoteolide 1a, b and c [18,19]. These compounds are also 20-membered marcolides which exhibit cytotoxic activity against several human tumor cell lines [20–22] and are produced by the marine symbiontic dinoflagellate *Amphidinium sp*. Iriomotolide 1a, 1b and 1c show remarkable cytotoxicity against B lymphocyte cells DG75 (IC_50_ = 0.0039 μM, 1.7 μM and 0.0038 μM) while amphidinolide A and U possess cytotoxic activities against murein lymphoma cells L1210 (IC_50_ = 3.7 μM and 10.7 μM) and against human epideromoid carcinoma cells (IC_50_ = 10.7 μM and 35.08 μM).

According to Skropeta (2008), polyketide metabolites have been reported from all water depths, but interestingly only 8% of the marine natural products known so far are produced by organisms obtained at depth greater than 1000 m [23]. As a matter of course, this might be due to the fact that the deep sea is hardly accessible. In the present study, it was shown by cultivation of strain M71-A77 with habitat sea water (38.6‰) that levantilides are also produced under the high salinity conditions occurring *in situ* in the Mediterranean Sea. Though strains of *Micromonospora spp.* were frequently isolated from deep sea habitats [24–26], to the best of our knowledge the levantilides are the first natural products described from a *Micromonospora* sp. strain isolated from the deep sea.

## 3. Experimental Section

### 3.1. Isolation and identification of strain M71-A77

Strain M71-A77 has been isolated from a sediment core (1.5–5 cm sediment horizon) from 4400 m depth during a research cruise with RV Meteor M71/2 in the Eastern Mediterranean Sea, the so-called Levantine Sea [34° 25.48 N, 26° 05.39 E]. One gram of the sediment sample was transferred to a sterile petri dish and dried for 2 months at 20 °C prior to incubation for 1 h at 120 °C dry heat. Sediment was then re-suspended in demineralized water and inoculated on agar plates of XJ4-medium containing of 1 L of demineralized water 18 g agar, 0.1 g histidine, 1 g raffinose, 0.5 g sodium hydrogen phosphate, 1.7 g potassium chloride, 0.05 g magnesium sulfate, 0.01 g iron sulfate, 0.02 g calcium carbonate, 0.5 mg thiamine hydrogen chloride, 0.5 mg riboflavine, 0.5 mg niacine, 0.5 mg piridoxin, 0.5 mg calcium pantothenate, 0.5 mg inositol, 0.5 mg para aminobenzoic acid and 0.25 mg biotin. After 2 months of incubation at 28 °C, strain M71-A77 was isolated by transferring to fresh XJ4-medium. The strain was classified by 16S rRNA gene sequence analysis according to Gärtner *et al.* [27]. The 16S rRNA gene sequence was deposited in the EMBL Nucleotide Sequence Database and was assigned the accession no. FR714833.

### 3.2. Chemical analysis

#### General experimental procedures

The optical rotation was measured on a Perkin Elmer model 241 polarimeter. UV-spectra were obtained on a NanoVue (GE Healthcare). NMR spectra were recorded on a Bruker DRX500 spectrometer (500 and 125 MHz for ^1^H and ^13^C NMR, respectively), using the signals of the residual solvent protons and the solvent carbons as internal references (*δ*_H_ 2.04 and *δ*_C_ 28.9 ppm for acetone-*d*_6_; *δ*_H_ 2.50 and *δ*_C_ 39.51 ppm for DMSO-*d*_6_). High-resolution mass spectra were acquired on a benchtop time-of-flight spectrometer (MicrOTOF, Bruker Daltonics) with positive electrospray ionization. Analytical reversed phase HPLC-UV/MS experiments were performed using a C_18_ column (Phenomenex Onyx Monolithic C18, 100 × 3.00 mm) applying an H_2_O (A)/MeCN (B) gradient with 0.1% HCOOH added to both solvents (gradient: 0 min 5% B, 4 min 60% B, 6 min 100% B; flow 2 mL/min) on a VWR Hitachi Elite LaChrom system coupled to an ESI-ion trap detector (Esquire 4000, Bruker Daltonics). Preparative HPLC was carried out using a Phenomenex Gemini C18 110A AXIA, 100 × 50.00 mm column.

#### Isolation of levantilides A and B

10 L of liquid starch-peptone medium (1L demineralized water 10 g starch, 5 g soja peptone, 15 g Tropic Marin® sea salt and 1 g calcium carbonate) were used for cultivation of strain M71-A77. After 8 days of incubation (28 °C, 125 rpm), the culture supernatant was separated from the cells by centrifugation at 10,000 rpm for 10 min (Beckman J2-MC). Cell pellets were suspended in methanol and homogenized three times with an Ultra Turax T25 basic (IKA-Werke GmbH & Co., Staufen, Germany) at 17.500 U/min for 1 min. After additional centrifugation, the methanol extract was decanted and dried. The culture broth supernatant was extracted with ethylacetate (1:1). The dried extracts were dissolved in methanol and analyzed by HPLC-UV/MS. Levantilides A and B were detected at 4.2 and 4.5 min with a maximum UV-absorption at 260 nm. For structure analysis, **1** and **2** were separated by reversed phase HPLC. For that purpose, HCOOH (0.1%) was added to the solvents H_2_O (A) and MeCN (B) and a gradient from 10% B over 60% B (reached after 17 min) to 100% B was applied (flow 15 mL/min). Levantilides A and B were detected at 16.6 and 17.8 min. Thus, 7 mg of **1** and 3 mg of **2** were obtained.

**Levantilide A (1):** colorless, amorphous solid; [α]^20^ d −72.4 (*c* 0.145, MeOH); UV (MeOH) λ_max_ (log ɛ) 262 (4.61); for 1D and 2D NMR data see Table 1 and SI; HRESIMS *m/z* 531.3676 [M + Na]^+^ (C_30_H_52_NaO_6_, 531.3656).

**Levantilide B (2):** colorless, amorphous solid; [α]^20^ d −97.5 (*c* 0.04, MeOH); UV (MeOH) λ_max_ 261 (log ɛ) (4.48); ^1^H NMR (acetone-*d*_6_, 500 MHz) δ 7.15 (1H, dd, *J* = 15.1, 11.0, H-3), 6.36 (1H, dd, *J* = 15.1, 11.0, H-4), 6.11 (1H, ddd, *J* = 15.1, 9.8, 4.4, H-5), 5.83 (1H, d, *J* = 15.1, H-2), 4.87 (1H, d, *J* = 8.0, H-15), 4.81 (1H, dt, *J* = 10.2, 2.5, H-19), 4.17 (1H, dt, *J* = 11.9, 3.1, H-9), 4.10 (1H, m, H-7), 3.18 (1H, m, H-11) 2.69 (1H, m, H-16), 2.66 (1H, m, H-6a), 2.46 (1H, m, H-6b), 2.45 (2H, m, H_2_-22), 2.42 (2H, q, *J* = 7.5, H_2_-24), 1.99 (1H, m, H-13a), 1.87 (1H, m, H-18), 1.86 (1H, m, H-10), 1.72 (1H, ddd, *J* = 15.0, 11.2, 3.3, H-8a), 1.69 (1H, dd, *J* = 13.1, 10.9, H-13b), 1.63 (3H, s, H_3_-28), 1.62 (1H, m, H-21a), 1.57 (1H, m, H-20a), 1.50 (1H, m, H-20b), 1.47 (1H, m, H-21b), 1.43 (1H, m, H-17a), 1.41 (1H, m, H-12), 1.34 (1H, ddd, J = 15.0, 5.4, 3.0, H-8b), 1.11 (1H, ddd, *J* = 14.6, 9.0, 5.1, H-17b), 0.98 (3H, d, *J*=7.0, H_3_-30), 0.96 (3H, t, *J* = 7.5, H_3_-25), 0.91 (3H, d, *J* = 7.5, H_3_-26), 0.87 (3H, d, *J* = 7.0, H_3_-27), 0.71 (3H, d, *J* = 7.0, H_3_-29); for ^13^C NMR data see Table 2; HRESIMS *m/z* 529.3509 [M + Na]^+^ (C_30_H_50_NaO_6_, 529.3500).

### 3.3. Production of levantilide A (1) and B (2) at in situ salinity

Strain M71-A77 was tested for the production of secondary metabolites at habitat salinity (38.6 ‰). For that purpose, Tropical Marine® salt and aqua dest. were replaced by Mediterranean Sea water obtained from the sampling site. After 8 days of cultivation at 28 °C, the culture broth was extracted with ethylacetate and analyzed by analytical HPLC-UV/MS as described above.

### 3.4. Antimicrobial tests

Antimicrobial activity of compound **1** and **2** (100 μM) was tested against the Gram-positive bacteria *Bacillus subtilis* (DSM 347)*, Staphylococcus lentus* (DSM 6672)*,* the Gram-negative bacteria *Xanthomonas campestris* (DSM 2405)*, Escherichia coli* (DSM 498)*, Erwinia amylovora* (DSM 50901)*, Pseudomonas fluorescens* (NCIMB 10586)*, Pseudomonas syringae* (DSM 50252)*, Ralstonia solanacearum* (DSM 9544), the yeast *Candida glabrata* (DSM 6425) and the fungus *Septoria tritici* as described by Lang *et al.* in 2007 [28]. The results were compared to a positive (100 μM chloramphenicol for bacteria and 100 μM cycloheximide for *C. glabrata* and *S. tritici*) and a negative (no compound) control on the same plate.

### 3.5. Cytotoxic tests

The *in vitro* antiproliferative activities of compound **1** against the gastric cancer cell line GXF 251L, lung cancer cell line LXFL 529L, melanoma cancer cell line MEXF 462NL, mammary cancer cell line MAXF 401NL, renal cancer cell line RXF 486L and pancreatic cancer cell line PAXF 1657L were determined by Oncotest GmbH (Freiburg, Germany) using a modified propidium iodide monolayer assay [29]. Compound **2** was not tested by Oncotest GmbH, for there was not enough left of the compound.

## Figures and Tables

**Table 1:** NMR spectroscopic data (500 MHz, DMSO-*d*_6_) of levantilide A (**1**).

levantilide A (1)					
position	δ_C_	δ_H_, *J* [Hz]	COSY	HMBC	NOESY
1	166.1, C				
2	120.4, CH	5.78, d (15.5)	3	1, 3, 4, 5	3, 4
3	143.8, CH	7.06, dd (11.2, 15.5)	2, 4	1, 2, 4, 5	2, 4, 5
4	130.6, CH	6.29, dd (11.2, 15.2)	3, 5	2, 3, 6	2, 3, 5, 6b
5	139.8, CH	6.08, ddd (4.4, 10, 15.2)	4, 6	3, 6, 7	3, 4, 6a, 7
6a	39.6, CH_2_	2.57, m	5, 6b, 7	4, 5, 7, 8	5
6b		2.33, dt (14.4, 9.9)	5, 6a, 7	4, 5, 7, 8	4
7	67.2, CH	3.98, m	6, 7-OH, 8		8b, 5
7-OH		4.81, br. d (3.8)	7	7, 8	
8a	33.4, CH_2_	1.51^a^, m	7, 8b, 9	6, 7, 9	8b
8b		1.16, ddd (14.6, 5.6, 2.3)	7, 8a, 9	6, 7, 9	7, 8a, 11
9	67.4, CH	3.92, br. d (11.2)	8, 9-OH, 10	30	12
9-OH		4.63, br. s	9	8, 9, 10	
10	41.0, CH	1.68, m	9, 11, 30		
11	75.3, CH	2.98 br. ddd (8.9, 6.0, 1.8)	10, 11-OH, 12	9, 10, 12, 13, 29, 30	8b, 29
11-OH		4.00, d (6.0)	11	10, 11, 12	
12	32.1, CH	1.24, m	11, 13, 29		9
13a	40.1, CH_2_	1.87, br. d (12.8)	12, 13b, 15, 29	11, 12, 14, 15, 27, 28, 29	13b
13b		1.51^a^, m	12, 13a, 15, 29	11, 12, 14, 15, 27, 28, 29	13a, 15
14	132.8, C				
15	132.7, CH	4.76, d (7.8)	13, 16	12, 13, 16, 17, 27, 28	13b, 17, 29
16	29.4, CH	2.59, m	15, 17, 27	14, 17, 27	27, 28
17a	40.9, CH_2_	1.34, m	16, 17b, 18	15, 16, 18, 19, 26, 27	17b
17b		1.05, ddd (13.5, 8.7, 5.0)	16, 17a, 18	15, 16, 18, 19, 26, 27	17a, 27
18	33.4, CH	1.75, m	17, 19, 26	16, 17, 19, 20, 26	
19	77.4, CH	4.71, dt (10, 2.4)	18, 20	1, 17, 20, 21,26	21, 26
20a	27.9, CH_2_	1.52^a^, m	19, 21		
20b		1.47^a^, m	19, 21		
21	22.0, CH_2_	1.30^b^, m	20		19
22	36.1, CH_2_	1.31^b^, m	23		23, 23-OH
23	70.8, CH	3.28, m	22, 23-OH, 24	21, 22, 25	22, 25, 26
23-OH		4.25, d (5.2)	23	22, 23, 24	22, 26
24a	29.7, CH_2_	1.30^b^, m	23, 25		25
24b		1.27^b^, m	23, 25		25
25	10.1, CH_3_	0.82, t (7.4)	24	23, 24	23, 24
26	17.5, CH_3_	0.88, d (6.2)	18	17, 18, 19	19, 23, 23-OH
27	21.5, CH_3_	0.84, d (6.8)	16	15, 16, 17	16, 17b
28	17.0, CH_3_	1.53^a^, s		13, 14, 15	16
29	17.7, CH_3_	0.58, d (6.7)	12	11, 12, 13	11, 15
30	11.1, CH_3_	0.86, d (5.9)	10	9, 10, 11	9, 11

a, b signals are overlapping.

**Table 2:** NMR spectroscopic data of the levantilides in acetone-*d*_6_ (500 MHz).

	levantilide A (1)		levantilide B (2)	
	
	δ_C_	δ_H_	δ_C_	δ_H_
1	166.9		166.9	
2	122.0	5.83	121.9	5.83
3	144.3	7.14	144.4	7.15
4	131.9	6.36	131.9	6.36
5	140.1	6.09	140.2	6.11
6a	40.6	2.69	40.6	2.66
6b		2.47		2.46
7	69.3	4.11	69.4	4.10
8a	33.8	1.73	33.9	1.72
8b		1.34		1.34
9	69.3	4.18	69.4	4.17
10	42.2	1.86	42.3	1.86
11	77.4	3.17	77.4	3.18
12	33.2	1.41	33.3	1.41
13a	41.4	2.00	41.4	1.99
13b		1.69		1.69
14	134.1		134.1	
15	134.1	4.86	134.1	4.87
16	30.7	2.70	30.7	2.69
17a	41.9	1.47	41.9	1.43
17b		1.12		1.11
18	34.7	1.87	34.7	1.87
19	78.8	4.83	78.6	4.81
20a	28.9	1.61	28.3	1.57
20b		1.53		1.50
21a	23.3	1.42	21.3	1.62
21b				1.47
22	37.6	1.42	42.1	2.45
23	72.7	3.42	210.5	
24a	31.1	1.43	36.0	2.42
24b		1.37		
25	10.4	0.90	8.0	0.96
26	18.6	0.91	18.4	0.91
27	22.0	0.86	21.8	0.87
28	17.6	1.62	17.6	1.63
29	18.5	0.70	18.4	0.71
30	10.8	0.98	11.0	0.98

## References

[1] Lam KS Discovery of novel metabolites from marine actinomycetes Curr Opin Microbiol 2006 9 245 251 16675289 10.1016/j.mib.2006.03.004

[2] Berdy J Bioactive microbial metabolites J Antibiot 2005 58 1 26 15813176 10.1038/ja.2005.1

[3] Jensen PR Mincer TJ Williams PG Fenical W Marine actinomycete diversity and natural product discovery Antonie van Leeuwenhoek 2005 87 43 48 15726290 10.1007/s10482-004-6540-1

[4] Fiedler HP Bruntner C Bull AT Ward AC Goodfellow M Potterat O Puder C Mihm G Marine actinomycetes as a source of novel secondary metabolites Antonie van Leeuwenhoek 2005 87 37 42 15726289 10.1007/s10482-004-6538-8

[5] Magarvey NA Keller JM Bernan V Dworkin M Sherman DH Isolation and characterization of novel marine-derived actinomycete taxa rich in bioactive metabolites Appl Environ Microbiol 2004 70 7520 7529 15574955 10.1128/AEM.70.12.7520-7529.2004 PMC535209

[6] Bernan VS Greenstein M Carter GT Mining marine microorganisms as a source of new antimicrobials and antifungals Curr Med Chem-Anti-Infect Agents 2004 3 181 195

[7] Hohmann C Schneider K Bruntner C Irran E Nicholson G Bull AT Jones AL Brown R Stach JEM Goodfellow M Caboxamycin, a new antibiotic of the benzoxazole family produced by the deep-sea strain Streptomyces sp. NTK 937* J Antibiot 2009 62 99 104 19198633 10.1038/ja.2008.24

[8] Hohmann C Schneider K Bruntner C Brown R Jones AL Goodfellow M Kramer M Imhoff JF Nicholson G Fiedler HP Albidopyrone, a new [alpha]-pyrone-containing metabolite from marine-derived Streptomyces sp. NTK 227* J Antibiot 2009 62 75 79 19132055 10.1038/ja.2008.15

[9] Thingstad TF Krom MD Mantoura RFC Flaten GAF Groom S Herut B Kress N Law CS Pasternak A Pitta P Nature of phosphorus limitation in the ultraoligotrophic Eastern Mediterranean Science 2005 309 1068 1071 16099984 10.1126/science.1112632

[10] Katz L Ashley GW Translation and protein synthesis: macrolides Chem Rev 2005 105 499 528 15700954 10.1021/cr030107f

[11] Ohta E Ohta S Kubota NK Suzuki M Ogawa T Yamasaki A Ikegami S Micromonospolide A, a new macrolide from Micromonospora sp Tetrahedron Lett 2001 42 4179 4181

[12] Weinstein MJ Wagman GH Marquez JA Testa RT Oden E Waitz JA Megalomicin, a new macrolide antibiotic complex produced by Micromonospora J Antibiot 1969 22 253 258 5810991 10.7164/antibiotics.22.253

[13] Hatano K Higashide E Shibata M Studies on juvenimicin, a new antibiotic I J Antibiot 1976 29 1163 1170 993103 10.7164/antibiotics.29.1163

[14] Satoi S Muto N Hayashi M Fujii T Otani M Mycinamicins, new macrolide antibiotics. I J Antibiot 1980 33 364 376 7410205 10.7164/antibiotics.33.364

[15] Waitz JA Drube CG Moss EL Weinstein MJ Biological studies with rosamicin, a new micomonospora-produced macrolide antibiotic J Antibiot 1972 25 647 652 4486485 10.7164/antibiotics.25.647

[16] Kobayashi J Ishibashi M Nakamura H Ohizumi Terufumi Y Amphidinolide-A, a novel antineoplastic macrolide from the marine dinoflagellate sp Tetrahedron Lett 1986 27 5755 5758

[17] Tsuda M Endo T Kobayashi J Amphidinolide U, novel 20-membered macrolide from marine dinoflagellate amphidinium sp Tetrahedron 1999 55 14565 14570

[18] Tsuda M Oguchi K Iwamoto R Okamoto Y Fukushi E Kawabata J Ozawa T Masuda A Kitaya Y Omasa K Iriomoteolide-1a, a potent cytotoxic 20-membered macrolide from a benthic dinoflagellate Amphidinium species J Org Chem 2007 72 4469 4474 17500570 10.1021/jo070414b

[19] Tsuda M Oguchi K Iwamoto R Okamoto Y Fukushi E Kawabata J Ozawa T Masuda A Iriomoteolides-1b and -1c, 20-membered macrolides from a marine dinoflagellate Amphidinium species J Nat Prod 2007 70 1661 1663 17927263 10.1021/np0702537

[20] Kobayashi J Tsuda M Amphidinolides, bioactive macrolides from symbiotic marine dinoflagellates Nat Prod Rep 2004 21 77 93 15039836 10.1039/b310427n

[21] Kobayashi J Ishibashi M Bioactive metabolites of symbiotic marine microorganisms Chem Rev 1993 93 1753 1769

[22] Kobayashi J Kubota T Bioactive macrolides and polyketides from marine dinoflagellates of the genus Amphidinium J Nat Prod 2007 70 451 460 17335244 10.1021/np0605844

[23] Skropeta D Deep-sea natural products Nat Prod Rep 2008 251131 1166 19030606 10.1039/b808743a

[24] Pathom-aree W Stach J Ward A Horikoshi K Bull A Goodfellow M Diversity of actinomycetes isolated from Challenger Deep sediment (10.898 m) from the Mariana Trench Extremophiles 2006 10 181 189 16538400 10.1007/s00792-005-0482-z

[25] Prieto-Davo A Fenical W Jensen PR Comparative actinomycete diversity in marine sediments Aquat Microb Ecol 2008 52 1 11

[26] Colquhoun JA Heald SC Li L Tamaoka J Kato C Horikoshi K Bull AT Taxonomy and biotransformation activities of some deep-sea actinomycetes Extremophiles 1998 2 269 277 9783174 10.1007/s007920050069

[27] Gärtner A Wiese J Imhoff JF *Amphritea atlantica gen.* nov., *sp.* nov., a gammaproteobacterium from the Logatchev hydrothermal vent field Int J Syst Evol Microbiol 2008 58 34 39 18175678 10.1099/ijs.0.65234-0

[28] Lang G Wiese J Schmaljohann R Imhoff JF New pentaenes from the sponge-derived marine fungus *Penicillium rugulosum*: structure determination and biosynthetic studies Tetrahedron 2007 63 11844 11849

[29] Dengler WA Schulte J Berger DP Mertelsmann R Fiebig HH Development of a propidium iodide fluorescence assay for proliferation and cytotoxicity assays Anti-Cancer Drugs 1995 6 10.1097/00001813-199508000-00005 7579556

